# Occupational inequalities and gender differences: work accidents, Brazil, 2019

**DOI:** 10.11606/s1518-8787.2024058005342

**Published:** 2024-04-11

**Authors:** Luciana de Melo Gomides, Mery Natali Silva Abreu, Ada Ávila Assunção

**Affiliations:** I Universidade Federal de Itajubá Instituto de Ciências Puras e Aplicadas Coordenação de Engenharia de Saúde e Segurança Itabira MG Brazil Universidade Federal de Itajubá. Instituto de Ciências Puras e Aplicadas. Coordenação de Engenharia de Saúde e Segurança. Itabira, MG, Brazil; II Universidade Federal de Minas Gerais Faculdade de Medicina Programa de Pós-Graduação em Saúde Pública Belo Horizonte MG Brazil Universidade Federal de Minas Gerais. Faculdade de Medicina. Programa de Pós-Graduação em Saúde Pública. Belo Horizonte, MG, Brazil; III Universidade Federal de Minas Gerais Escola de Enfermagem Departamento de Enfermagem Aplicada Belo Horizonte MG Brazil Universidade Federal de Minas Gerais. Escola de Enfermagem. Departamento de Enfermagem Aplicada. Belo Horizonte, MG, Brazil; IV Universidade Federal de Minas Gerais Faculdade de Medicina Departamento de Medicina Preventiva e Social Belo Horizonte MG Brazil Universidade Federal de Minas Gerais. Faculdade de Medicina. Departamento de Medicina Preventiva e Social. Belo Horizonte, MG, Brazil

**Keywords:** Accidents, Occupational, Gender, Sociodemographic Factors, Risk Factors, Health Surveys

## Abstract

**OBJECTIVE:**

To analyze the distribution and association of sociodemographic and occupational factors with self-reported work accidents (WA) in a representative sample of the Brazilian population, with emphasis on occupational class, and to examine gender differences in this distribution.

**METHODS:**

A population-based cross-sectional study, using data from the 2019 National Health Survey (PNS), analyzed the responses of a sample of adults aged 18 or over. Factors associated with WA were investigated using binary logistic regression and hierarchical analysis using blocks (sociodemographic and occupational variables). The final model was adjusted by variables from all blocks, adopting a significance level of 5%. The values of odds ratios (OR) and respective confidence intervals were obtained.

**RESULTS:**

Among the participants, 2.69% reported having suffered a WA, with a higher prevalence in men (3.37%; 95%CI 2.97–3.82%) than in women (1.86%; 95%CI 1.55–2.23%). The analysis identified that age group, night work, working hours, and exposure to occupational risks were associated with WA, with emphasis on gender differences. The class of manual workers, both qualified (OR_women_ = 2.87; 95%CI 1.33–6.21 and OR_men_ = 2.46; 95%CI 1.37–4.40) and unskilled (OR_women_ = 2.55; 95%CI 1.44–4.50 and OR_men_ = 3.70; 95%CI 1.95–7.03), had a higher chance of WA than the class of managers/professionals.

**CONCLUSION:**

Occupational factors contributed significantly to the increase in the probability of WA for men and women, with greater magnitude among those positioned in the lower strata of the occupational structure. The results obtained are clues for working out WA prevention actions.

## INTRODUCTION

There is a convergence between schooling, income, and the place occupied by the individual in the occupational structure, which reflects on the type, outcome and intensity of the activities carried out by male and female workers. Determined by economic and political processes, the occupational structure represents the idea of a society that hierarchizes and discriminates between occupations^1^. It is known that, in the lower classes of the structure, mortality rates are generally higher, as well as worse health outcomes, compared to classes positioned on the upper floors^2,3^. The nature of the activities and the conditions under which the work is carried out are specific to each class, explaining, at least in part, health inequalities^4,5^.

Firstly, access to employment depends on the level of education^2^. Secondly, in occupations with lower education/qualification requirements, individuals have a greater chance of being exposed to unhealthy and dangerous environments, culminating in a higher prevalence of work accidents (WA)^6,7^. Thirdly, previous results showed that the effects of risks are not identical when comparing occupational groups^6^.

WA are sudden events, resulting from unnatural causes, that occur in the work environment during the exercise of activities by the worker, causing bodily injuries or functional disturbances. Considered a public health problem, they are a source of morbidity and mortality and can lead to work incapacity and early exit from the workforce^4,8^. In addition to pain and suffering for the victims, these events generate social security costs, health service expenses, and social and financial burdens for society as a whole^9,10^.

Within the scope of investigations into the distribution of WA based on occupational structure, research brings enlightening perspectives. Although recent results in Brazil focus on local studies^9,11^, many targeting specific categories^12^, a more comprehensive view is possible.

A more complete approach to inequalities in WA requires the incorporation of gender differences in the analyses, so that it is important to clarify the connection between sex and gender, as suggested by authors who study the links between work and health^13^. Sex concerns biological factors, including chromosomal and hormonal. In turn, gender refers to the expectations and socially constructed roles considered appropriate to men or women^14^. This type of role assignment shapes experiences and inequalities in access to resources and opportunities. For example, the distribution between men and women of time allocated to work and domestic activities remains unbalanced, despite the increase in women’s participation in the labor market^15^.

In this sense, this study aims to analyze the distribution and association of sociodemographic and occupational factors with self-reported work accidents in a representative sample of the Brazilian population, with an emphasis on occupational class, as well as examining gender differences in this distribution.

## METHODS

### Study Design, Data Source and Sample Population

The cross-sectional study analyzed secondary data from the 2019 National Health Survey (PNS)^16,17^. PNS probabilistic sampling, representative of the Brazilian population aged 15 or over, was carried out through a three-stage conglomerate process, including stratification of primary sampling units. In the first stage, the census sectors, or sets of sectors constituting the primary sampling units, were selected. In the second stage, households were selected. For the third stage, in each household, one resident was randomly selected to answer the individual questionnaire. Details about the sampling process, design and implementation of the PNS 2019 are published^16^.

For the analyses of this study, employed people (among the selected residents) aged 18 years or over, who answered the questionnaire module referring to the characteristics of work and social support (Module M), were considered eligible. Military personnel, employers, and people who take care of domestic tasks at home or for people close to them were excluded. After removing those who were ineligible, a total sample of 50,056 individuals was obtained who answered all the questions of interest in this research.

### Study Variables

The outcome variable WA, of the dichotomous type, was obtained through the answer to question O21, of module O of the PNS 2019, “In the last twelve months, have you been involved in an accident at work (without considering traffic accidents and/or in commuting to work)?”

The first block of independent variables concerns the following sociodemographic factors: sex (man, woman), age group (18–29 years, 30–39 years, 40–49 years, 50–59 years, and ≥ 60 years), level of education (higher, secondary, elementary, and no schooling), race/color (white, black, mixed, and others). Occupational factors constitute the second block, as follows: night work (yes, no), weekly working hours (less than 40 hours, between 40 and 44 hours, and over 44 hours), exposure to occupational risks, i.e. exposure to chemical, physical, or biological risks at work (yes, no). Occupational classes were defined based on the answer to question E12 “What was your occupation (position or function) in that job?”.

Classification of occupations is a method that allows organizing occupational groups defined according to income, education, type, and demand of the tasks performed. Indicators based on occupational classes capture the effects of these factors on workers’ health and safety^2,6,18^. The *Classificação Brasileira de Ocupações* (CBO – Brazilian Classification of Occupations)^19^is in line with the International Standard Classification of Occupations (ISCO) prepared by the International Labor Organization (ILO)^20^to facilitate the production of information about employment and workers in different countries. The classification criteria and terminology used at ISCO to define and name occupations are widely discussed and publicized, in order to guide decision-making and the development of specific actions and programs, as well as support research^20^. The current version of the ISCO considers the level of competence as the basic criterion to define the system that incorporates large occupational groups, main subgroups, and base subgroups and groups (occupational families). In this context, the CBO defines competence as “a function of the complexity, breadth and responsibility of the activities carried out in employment or another type of employment relationship”^19^. The assessment of a competence is operational, as it considers the nature of the work specific to that occupation, the education required to perform that task, and the specific training or previous experience, which would promote the development of the relevant skills^20^.

The alternative answers about occupation that appear in the PNS questionnaire are based on the Classification of Occupations for Household Surveys (COD), which is an adaptation of the CBO for household surveys by the Brazilian Institute of Geography and Statistics (IBGE)^21^.

In this study, the composition of six categories (Chart) was used, representing the ten major occupational groups of the COD. This strategy has the advantage of avoiding an excess of variables that would cause problems in the estimates of statistical studies^3^. This composition was suggested in the Socioeconomic Status of Occupations Scale, developed by Pastore e Silva^22^, which is widespread in Brazil as it combines individual educational and economic positions within a specific occupational class. In this typology, the distinction between manual and non-manual work is an attempt to capture the discrimination resulting from the social structure and the degree of prestige granted to different professions^1,3,22^.

**Chart:** Organization of occupations according to the occupational composition of Pastore and Silva^19^ in relation to the major occupational groups and subgroups of the Classification of Occupations for Household Surveys (COD) of the Brazilian Institute of Geography and Statistics (IBGE)^18^.

Occupational composition^19^	IBGE COD Large groups (GG)	IBGE COD Subgroups (SG)
Managers ans high-level professionals (High)	GG00 – Armed forces professionals, military firefighters	
	GG01 – Directors and managers	SG11 – Executive directors, public administration directors and member of the executive branch
		SG12 – Administrative and commercial managers
		SG13 – Production and operation directors and managers
		SG14 – Managers of hotels, restaurants, shops and other services
	GG02 – Science professionals and intellectuals	SG21 – Science and engineering professionals
		SG22 – Health professionals
		SG23 – Teaching professionals
		SG24 – Specialist in administration organization
		SG25 – Information and communications technology professionals
		SG26 – Professionals in law, social, and cultural sciences
Technicians (Upper-middle)	GG03 – Technicians and midlevel professionals	SG31 – Mid-level science and engineering professionals
		SG 32 – mid-level healthcare and related professionals
		SG 33 – Mid-level professionals in financial and administrative operations
		SG34 – Mid-level professionals in legal, social, cultural, and related services
		SG35 – Mid-level information and communications technology technicians
Routine non-manual workers (Middle-middle)	GG04 – Administrative support workers	SG41 – Clerks
		SG42 – Direct customer service workers
		SG43 – Numerical calculation workers and those responsible for recording materials
		SG44 – Other administrative service workers
	GG05 – Service workers, commercial vendors, and markets	SG51 – Personal service workers
		SG52 – Salespeople
		SG53 – Personal care workers
		SG54 – Protection and security services workers
Skilled manual workers (Lower-middle)	GG07 – Skilled workers, laborers, and artisans in construction, mechanical arts, and other trades	SG71 – Skilled workers and construction workers, including electricians
		SG72 – Qualified workers and workers in metallurgy, mechanical construction, and similar areas
		SG73 – Craftsmen and graphic arts workers
		SG74 – Workers specialized in electricity and electronics
		SG75 – Wood, clothing, and related food processing workers and officers
	GG08 – Plant and machine operators and assemblers	SG 81 – Operators of stationary facilities and machinery
		SG82 – Assemblers
		SG83 – Drivers of vehicles and operators of heavy mobile equipment
Unskilled manual workers (Upper-lower)	GG09 – Elementary workers	SG91 – Domestic workers and other workers cleaning the interior of buildings
		SG93 – Basic workers in mining, construction, manufacturing, and transport
		SG94 – Food preparation helpers
		SG95 – Street vendors and similar workers
		SG96 – Garbage collectors and other
Rural workers (Lower-lower)	GG09 – Elementary workers	elementary occupations
	GG06 – Qualified agricultural, forestry, hunting, and fishing workers	SG61 – Qualified agricultural farmers
		SG62 – Skilled forestry workers and hunters

### Statistical analysis

All WA analyses were carried out with attention to the characteristics of the complex PNS 2019 sample. Thus, the expansion factors or sample weights of the households and all their residents, as well as the resident selected for the interview, were considered. All calculations were performed with the help of Stata 16.0 software. First, a descriptive analysis of the sample was carried out with all variables of interest. Differences in proportions were estimated using Pearson’s chi-square test, considering a p-value < 0.05. Prevalence was calculated with respective 95% confidence intervals (95%CI). The association between self-reported work accidents and independent variables was analyzed using binary logistic regression. The unadjusted and adjusted analyses were performed with a sample stratified by sex.

All variables with a p-value lower than 0.20 from the unadjusted analysis were included in the adjusted analysis. For the multivariable model, a hierarchical model was established with the inclusion of two different blocks: the first composed of sociodemographic variables and the second, occupational variables. The backward method was used for selecting variables, with only those significant at the 5% level remaining at the end. The magnitude of the association was estimated using the odds ratio (OR) with the respective 95%CI in all phases of the analysis.

The goodness of fit of the models was assessed using the Hosmer-Lemeshow test. Possible interactions between the variables that remained in the final model were tested. After defining the models, predictive calculations by occupational class were carried out for men and women, according to two distinct profiles: the first composed of characteristics with a greater chance of WA and the second with characteristics with a lower chance.

## RESULTS

Two million and forty-eight thousand Brazilian workers aged 18 or over reported having suffered a WA from 2018 to 2019, which corresponds to 2.69% (95%CI 2.42–2.98%) of the analyzed sample. The highest prevalence was observed among men compared to women, 3.37% (95%CI 2.97–3.82%) and 1.86% (95%CI 1.55–2.23%), respectively. Differences in the prevalence of WA were observed for the variables, according to gender (Table 1).

**Table 1:** Proportions of men and women and prevalence of work accidents stratified by sex, according to sociodemographic and occupational characteristics. National Health Survey, Brazil, 2019.

Variables	Men			Women			p-value^a^
	
	(n_exp_ = 50,541,462)			(n_exp_ = 41,991,625)			
	
	n_exp_/1000	%	Prevalence of accidents	n_exp_/1000	%	Prevalence of accidents	
	
			(95%CI)			(95%CI)	
Work accident							< 0.001
No	48,837.17	96.63	-	41,211.19	98.14		
Yes	1,704.30	3.37	3.37 (2.97–3.82)	780.43	1.86	1.86 (1.55–2.23)	
Age range (years)							< 0.001
≥ 60	12,244.81	9.15	2.16 (1.48–3.13)	9,624.72	7.86	0.58 (0.34–1.01)	
50–59	9,453.33	18.7	2.90 (2.16–3.87)	7,234.05	17.23	2.02 (1.39–2.94)	
40–49	10,767.98	21.31	3.13 (2.18–4.47)	10,304.56	24.54	2.26 (1.71–2.98)	
30–39	13,450.26	26.61	3.50 (2.79–4.39)	11,526.14	27.45	1.47 (1.06–2.04)	
18–29	4,625.08	24.23	4.27 (3.37–5.39)	3,302.15	22.92	2.21 (1.40–3.48)	
Race/color							< 0.001
White	20,971.49	41.49	2.75 (2.24–3.38)	18,866.19	44.93	1.55 (1.11–2.17)	
Black	6,107.36	12.08	4.41 (3.28–5.91)	5,138.69	12.24	2.25 (1.30–3.86)	
Mixed	22,649.09	44.81	3.74 (3.10–4.51)	17,465.47	44.81	2.11 (1.67–2.66)	
Others^b^	813.52	1.61	1.21 (0.59–2.45)	521.27	1.24	0.67 (0.25–1.76)	
Education level							< 0.001
Higher	7,583.61	15	1.22 (0.74–1.99)	10,238.29	24.38	1.17 (0.79–1.72)	
Secondary	19,026.78	37.65	2.95 (2.43–3.58)	17,560.47	41.82	2.29 (1.71–3.06)	
Primary	8,450.14	16.72	4.52 (3.15–6.45)	5,255.97	12.52	1.94 (1.34–2.79)	
No schooling	15,480.93	30.63	4.32 (3.62–5.14)	8,936.90	21.28	1.75 (1.26–2.43)	
Occupational class							< 0.001
Managers/professionals	5,682.31	11.24	1.01 (0.60–1.71)	7,329.41	17.45	0.97 (0.62–1.51)	
Technical	4,194.52	8.3	1.52 (1.01–2.29)	3,340.87	7.96	2.18 (1.38–3.43)	
No routine manual	11,527.50	22.81	2.05 (1.46–2.85)	17,316.08	41.24	1.62 (1.16–2.26)	
Qualified manual	16,757.40	33.16	3.9 (3.27–4.64)	3,639.55	8.67	3.00 (1.60–5.55)	
Rural	6,675.94	13.21	5.44 (3.86–7.62)	1,643.65	3.91	1.66 (0.97–2.84)	
Unqualified manual	5,703.79	11.29	5.80 (4.24–7.88)	8,722.08	20.77	2.51 (1.83–3.45)	
Night work							< 0.001
No	42,496.53	84.08	3.2 (2.77–3.69)	37,930.93	90.33	1.72 (1.40–2.10)	
Yes	8,044.93	15.92	4.29 (3.34–5.50)	4,060.70	9.67	3.17 (2.17–4.61)	
Weekly working hours							< 0.001
< 40	25,314.06	20.54	1.96 (1.56–2.46)	27,766.83	39.24	1.26 (0.89–1.77)	
40–44	10,861.41	51.04	3.34 (2.86–3.90)	6,887.88	43.29	2.23 (1.69–2.94)	
> 44	14,365.99	28.42	4.46 (3.50–5.66)	7,336.92	17.47	2.28 (1.71–3.02)	
Exposure to occupational risk							< 0.001
No	19,932.25	39.44	1.12 (0.81–1.54)	27,014.29	64.33	0.91 (0.69–1.19)	
Yes	30,609.22	60.56	4.84 (4.22–5.54)	14,977.33	35.67	3.57 (2.83–4.49)	

N_exp_: expanded number; 95%CI: 95% confidence interval.

Note: percentage by gender of the sample: men, 54.62% (95%CI 53.76–55.47%) and women 45.38% (95%CI 44.53–46.24%).

^a^ Associated with the equality of proportions test based on Pearson’s chi-square statistic.

^b^ Others: people who declared themselves yellow and indigenous.

In the unadjusted analysis, all independent variables were maintained (p < 0.20) and, therefore, taken for adjusted analysis for both sexes (Tables 2 and 3). In the final model adjusted for women, the variables age group, occupational classes, night work, working hours, and exposure to occupational risks remained associated with the outcome (Table 2). Greater chances of WA were observed among workers positioned in the qualified manual class (OR = 2.87; 95%CI 1.33–6.21) as compared to the class of managers and professionals. Greater chances of WA were found among women with self-reported working hours between 40 and 44 hours per week (OR = 1.87; 95%CI 1.20–2.90) and working hours greater than 44 hours per week (OR = 1.58; 95%CI 1.00–2.49), compared to women who self-reported working less than 40 hours per week. The model identified greater chances of WA among those who reported working night shifts (OR = 1.59; 95%CI 1.00–2.56). It is worth highlighting the interaction between age group and exposure to occupational risks, causing the need to produce an additional model to better discriminate the results (Table 4).

**Table 2:** Unadjusted and adjusted estimates of odds ratios for work accidents, among women, associated with sociodemographic and occupational characteristics, National Health Survey, Brazil, 2019.

Variables	n_exp_/1000	Not adjusted		Adjusted		
	
		OR	p-value	Block 1 Sociodemographic	Block 2 Occupational	Final model (Block 1+2)
		
				OR (95%CI)	OR (95%CI)	OR (95%CI)
Age range (years)						
≥ 60	9,624.72	Ref.		Ref.	-	-
50–59	7,234.05	3.53	< 0.001	3.56 (1.81–6.97)	-	-
40–49	10,304.56	3.95	< 0.001	3.95 (2.11–7.41)	-	-
30–39	11,526.14	2.55	0.005	2.54 (1.30–4.96)	-	-
18–29	3,302.15	3.86	< 0.001	3.54 (1.73–7.24)	-	-
Race/color						
White	44.93	Ref.		-	-	-
Black	12.24	1.46	0.268	-	-	-
Mixed	44.81	1.36	0.142	-	-	-
Others^a^	1.24	0.43	0.108	-	-	-
Education level						
Higher	10,238.29	Ref.		Ref.	-	-
Secondary	17,560.47	1.98	0.007	1.94 (1.21–3.12)	-	-
Primary	5,255.97	1.67	0.064	1.64 (0.95–2.83)	-	-
No schooling	8,936.90	1.5	0.121	1.57 (0.93–2.64)	-	-
Occupational class						
Managers/professionals	7,329.41	Ref.		-	Ref.	Ref.
Technical	3,340.87	2.27	0.012	-	1.60 (0.84–3.03)	1.64 (0.86–3.11)
No routine manual	17,316.08	1.68	0.068	-	1.74 (0.98–3.07)	1.71 (0.98–2.98)
Qualified manual	36.39.55	3.15	0.004	-	2.86 (1.32–6.20)	2.87 (1.33–6.21)
Rural	1,643.65	1.72	0.133	-	1.26 (0.60–2.63)	1.36 (0.65–2.86)
Unqualified manual	8,722.08	2.63	0.001	-	2.48 (1.40–4.38)	2.55 (1.44–4.50)
Night work						
No	37,930.93	Ref.		-	Ref.	Ref.
Yes	4,060.70	1.87	0.005	-	1.60 (1.00–2.53)	1.59 (1.00–2.56)
Weekly working hours						
< 40	27,766.83	Ref.		-	Ref.	Ref.
40–44	6,887.88	1.8	0.01	-	1.85 (1.19–2.86)	1.87 (1.20–2.90)
> 44	7,336.92	1.83	0.008	-	1.60 (1.02–2.53)	1.58 (1.00–2.49)
Exposure to occupational risk						
No	27,014.29	Ref.		-	Ref.	-
Yes	14,977.33	4.03	< 0.001	-	3.79 (2.58–5.58)	-
Age group (years) #work risks						
50–59 #risks (yes)	2,445.40	-	-	-	-	-
40–49 #risks (yes)	4,016.07	-	-	-	-	-
30–39 #risks (yes)	4,452.58	-	-	-	-	-
18–29 #risks (yes)	908.36	-	-	-	-	-

Ref.: reference category; OR: odds ratio; 95%CI: 95% confidence interval; n_exp_ = expanded number.

P-value of the final model Hosmer-Lemeshow test: 0.96.

^a^Others: people who declared themselves yellow and indigenous.

**Table 4:** Multivariate analysis evaluating factors associated with risky use for WA in women, stratifying by exposure to occupational risk, according to PNS, 2019.

Variables	OR (95%CI)	

	Women without exposure to occupational risk	Women exposed to occupational risk
Age range (years)		
≥ 60	Ref.	Ref.
50–59	2.83 (1.16–6.93)	3.40 (1.28–9.00)
40–49	3.53 (1.38–9.05)	3.19 (1.31–7.80)
30–39	1.91 (0.63–5.73)	2.25 (0.92–5.51)
18–29	1.06 (0.39–2.91)	5.55 (2.16–14.30)
Occupational class		
Managers/professionals	Ref.	Ref.
Technical	1.45 (0.41–5.17)	1.62 (0.80–3.28)
No routine manual	1.87 (0.70–4.98)	1.62 (0.83–3.13)
Qualified manual	1.92 (0.45–8.19)	3.31 (1.40–7.84)
Rural	3.71 (0.75–1.83)	1.07 (0.50–2.26)
Unqualified manual	2.81 (0.98–8.05)	2.42 (1.28–4.58)
Night work		
No	Ref.	Ref.
Yes	0.88 (0.38–2.05)	1.84 (1.05–3.24)
Weekly working hours		
< 4	Ref.	Ref.
40–44	2.17 (1.12–4.21)	1.72 (0.97–3.06)
> 44	2.19 (0.98–4.91)	1.35 (0.78–2.34)

WA: work accident; OR: odds ratio; 95%CI: 95% confidence interval; Ref.: reference category.

The additional model (Table 4), stratified by exposure to occupational risks, shows that the group of exposed women has a greater chance of WA among those aged between 18 and 29 years (OR = 5.55; 95%CI 2.16– 14,30), when compared to women over 60 years of age.

Among men, the final adjusted model (Table 3) indicated a greater chance of WA in the group of unskilled manual workers (OR = 3.70; 95%CI: 1.95–7.03), compared to the reference group. It was observed that the increase in the length of the working day, in the group of men, is related to an increased chance of WA. Self-reported night shift work (OR = 1.47; 95%CI 1.08–2.00) and exposure to occupational risks (OR = 3.37; 95%CI: 2.40–4.74) were associated with the outcome. A greater chance of WA was observed in the group of workers aged between 18 and 29 years (OR = 1.84; 95%CI 1.15–2.94), compared to the group aged over 60 years. The models for both sexes showed good adjustment, according to the Hosmer-Lemeshow statistic (p > 0.05).

**Table 3:** Unadjusted and adjusted estimates of odds ratios for work accidents, among men, associated with sociodemographic and occupational characteristics, National Health Survey, Brazil, 2019.

Variables	n_exp_/1000	Not adjusted		Adjusted		
	
		OR	p-value	Block 1 Sociodemographic	Block 2 Occupational	Final model (Block 1+2)
		
				OR (95%CI)	OR (95%CI)	OR (95%CI)
Age range (years)						
≥ 60	12,244.81	Ref.		Ref.		Ref.
50–59	9,453.33	1.35	0.224	1.41 (0.87–2.29)		1.19 (0.73–1.95)
40–49	10,767.98	1.46	0.158	1.61 (0.96–2.71)		1.25 (0.73–2.17)
30–39	13,450.26	1.65	0.028	2.01 (1.27–3.20)		1.42 (0.91–2.23)
18–29	4,625.08	2.02	0.003	2.37 (1.47–3.80)		1.84 (1.15–2.94)
Race/color						
White	20,971.49	Ref.		-		-
Black	6,107.36	1.63	0.007	-		-
Mixed	22,649.09	1.37	0.034	-		-
Others^a^	813.52	0.43	0.027	-		-
Education level						
Higher	7,583.61	Ref.		Ref.		-
Secondary	19,026.78	2.47	0.001	2.27 (1.32–3.88)		-
Primary	8,450.14	3.84	< 0.001	3.57 (1.88–6.78)		-
No schooling	15,480.93	3.66	< 0.001	4.01 (2.32–6.95)		-
Occupational class						
Managers/professionals	5,682.31	Ref.			Ref.	Ref.
Technical	4,194.52	1.51	0.233		1.21 (0.61–2.40)	1.17 (0.59–2.33)
No routine manual	11,527.50	2.04	0.027		1.89 (1.00–3.56)	1.80 (0.96–3.37)
Qualified manual	16,757.40	3.97	< 0.001		2.52 (1.40–4.52)	2.46 (1.37–4.40)
Rural	6,675.94	5.64	< 0.001		3.64 (1.88–7.07)	3.63 (1.87–7.04)
Unqualified manual	5,703.79	6.02	< 0.001		4.03 (2.12–7.70)	3.70 (1.95–7.03)
Night work						
No	42,496.53	Ref.			Ref.	Ref.
Yes	8,044.93	1.36	0.044		1.50 (1.10–2.04)	1.47 (1.08–2.00)
Weekly working hours						
< 40	25,314.06	Ref.			Ref.	Ref.
40–44	10,861.41	1.73	< 0.001		1.74 (1.31–2.31)	1.73 (1.31–2.30)
> 44 hours	14,365.99	2.33	< 0.001		2.17 (1.48–3.17)	2.19 (1.51–3.20)
Exposure to occupational risk						
No	19,932.25	Ref.			Ref.	Ref.
Yes	30,609.22	4.49	< 0.001		3.36 (2.38–4.74)	3.37 (2.40–4.74)

OR: odds ratio; 95%CI: 95% confidence interval; Ref.: reference category; n_exp_ = expanded number.

Hosmer-Lemeshow test p-value: 0.93.

^a^Others: people who declared themselves yellow and indigenous.

The probabilities of WA for the occupational classes of the group of women were analyzed (Figure A) according to the following profiles: 1) age 60 years or more without exposure to risks in the work environment, no report of working at night, working hours less than 40 hours per week; 2) age between 18 and 29 years old with exposure to risks in the work environment, self-report of working at night, working hours between 40 and 44 hours per week. For female managers or professionals in profile 1, the probability of reporting accidents was 0.14% (95%CI 0.01–0.28%), whereas for profile 2, the probability was 5.95% (95%CI 1.97–9.93%), even if they are included in the same occupational class. In profile 1, the probabilities of the outcome among women in the qualified manual, rural and unskilled manual classes were, respectively, 0.41% (95%CI 0.02–0.89%), 0.19% (95%CI 0.00–0.39%) and 0.36% (95%CI 0.05–0.68%). When evaluating women with profile 2, the probability of WA increased by 15.36% in the qualified manual class (95%CI 4.30–26.42%), 7.93% (95%CI 1.85–14.01%) in the rural class, and 13.87% (95%CI 5.21–22.52%) in the unskilled manual class (Figure A), compared to women in profile 1.

**Figure:**
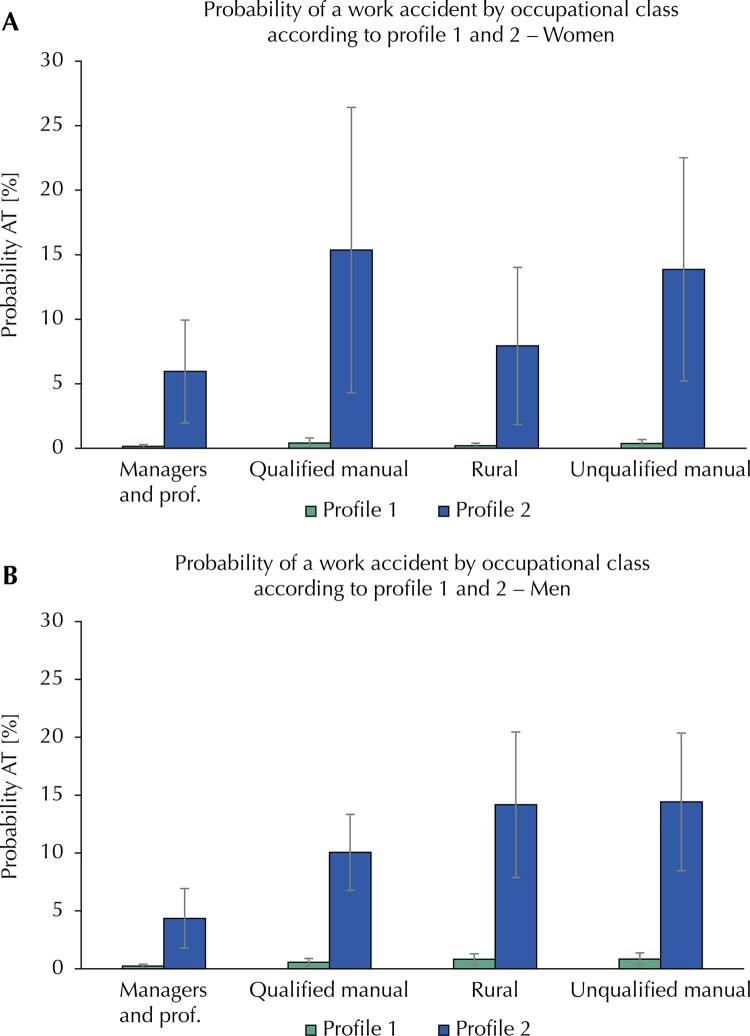
Estimated probability of a work accident for women (A) and men (B) according to occupational classes. National Health Survey, Brazil, 2019

WA: work accident.

In relation to occupational classes (Figure B), the following profiles were studied for men: 1) age 60 years or more, no report of working at night, working hours of less than 40 hours per week, and no exposure to occupational risks; 2) age group between 18 and 29 years old, self-report of working at night, working more than 44 hours per week, and exposure to occupational risks. For male managers or professionals in profile 1, the probability of reporting accidents was 0.23% (95%CI 0.06–0.39%), while in profile 2, included in the same class, the probability was 4.36% (95%CI 1.79–6.92%). The probability of WA among men in the skilled manual, rural and unskilled manual classes of profile 1 was 0.56% (95%CI 0.22–0.90%), 0.82% (95%CI 0.35–1 .29%), and 0.84% (95%CI 0.31–1.37%), respectively. When evaluating profile 2 men, the probability of WA increased by 10.06% (95%CI 6.78–13.34%) in the qualified manual class, 14.17% (95%CI 7.88–20.86%) in the rural class, and 14.42% (95%CI 8.48–20.36%) in the unskilled manual class (Figure B), compared to profile 1 men.

## DISCUSSION

To our knowledge, this is the first time, results are presented regarding the Brazilian population on the chance of WA in men and women, according to occupational classes. Following the global trend^23^, a decrease in the prevalence of WA was observed in 2019, compared to the previous edition of the PNS^10^.

Furthermore, there is a greater chance of WA in men and women in occupations classified as predominantly manual. These occupations are characterized by tasks with routine or elementary content; that is, repetitive and predefined activities, for which there is no requirement for specialized qualifications. According to the aforementioned standardized classifications, predominantly manual occupations are positioned at the bottom of the occupational hierarchy^1,19^. Workers in these occupations are generally less educated, rarely trained in topics related to occupational protection and safety, and have less power to avoid exposure to WA risks^7^.

Consistent with the literature, younger respondents were more likely to report WA. Firstly, because the job market selects individuals with greater muscular capacity and sensory ability to occupy the most insecure and unhealthy positions. Those who have not suffered the effects of work exposure or the adverse events of life, generally younger, will have more opportunities in this type of selection process^24,25^. The so-called healthy worker effect expresses this selection bias, that is, those who lost the ability to face the demands of strength, vigilance, and agility did not “survive” in the job market, as they are sick or retired earlier^25^. Secondly, younger people have not yet had enough experience to develop self-protection skills, explaining, at least in part, the greater likelihood of WA in this age group^26^.

The results regarding long hours and night work were statistically significant for both sexes. The mechanisms through which these factors negatively affect occupational health and safety are well documented^27^. Changes in cognitive functions and increased fatigue are effects of circadian cycle disturbances, which occur when people work at night and are also expected when recovery time is reduced due to long working hours. These effects are conditions at the origin of WA^28^.

Analysis of the probabilities of WA in occupational classes shows the significant association of age and occupational factors on the occurrence of these events. It was observed that younger age, exposure to occupational risks, night work, and long working hours, when combined, contribute to a significant increase in the likelihood of WA in all occupational classes, both for men and women.

The differences in the probabilities of WA between the different classes stood out, especially in the male group, when profile 2 was evaluated. The classes of rural and unskilled manual workers, subject to conditions associated with a greater likelihood of WA, presented significantly higher percentages compared to the classes of managers and professionals when exposed to the same set of factors. This result is consistent with the initial hypothesis of this study.

The observed gender differences are subject to interpretation^14^, indicating elements for further investigation. It is known that occupational exposures can vary when women are compared to men in the same class, because, generally, women perform tasks that are not assigned to men within the same occupation^29^. For example, in hospital establishments, male nursing assistants are exposed to tasks that require moving loads (moving patients in bed, etc.), with a greater chance of falling or twisting the trunk, and female assistants are responsible for procedures that require more dexterity, such as packaging materials or preparing medications, with a greater chance of pain in the upper limbs^5,29^.

Even with the increase in the number of women in the labor market in recent decades in industrialized countries, gender norms continue to structure the sexual division of labor^5,14,29^. Domestic chores and family care are roles that are unevenly distributed in society. In the case of women, the effects of the risks experienced during the working hours are compounded by the losses of restricted recovery time, among other problems related to the time dedicated to domestic work. Although historically underrepresented in jobs considered more dangerous, women have come to occupy positions previously dominated by men, for example, civil construction^30^. This current reality may be a hypothesis to explain the increased probability of WA when women are exposed to occupational risks that are well defined in heavily male sectors. In these cases, in addition to the occupational risks specific to each productive sector, gender stereotypes in the occupational environment weighed heavily on the female workers. In other words, the aforementioned stereotypes would explain both the lesser power to interfere in their own environment and the lesser access of women to management positions^31^. Finally, it is worth mentioning that gender research warns about the strongly masculine design of facilities and equipment, without considering the biological specificities of sex^5,29^.

The interpretations of the results obtained face some limitations. Memory bias may have caused underestimation of the results. However, the outcome is considered less sensitive to forgetting. Psychosocial factors, health conditions, and lifestyle habits, which may influence the chance of WA, were not addressed. However, there is evidence that, when related to WA, the contribution of these factors is limited to explaining the discrepancies observed when compared to occupational characteristics^6,32^. A high prevalence of WA was observed among men from the rural class. However, the result did not reach statistical significance among women in this class, making an in-depth analysis unfeasible. It is worth highlighting a possible representativeness bias, since the small number of WAs observed in the group of female rural workers may have influenced the power of the study to identify significant associations in this occupational class.

Gender differences in the likelihood of WA by occupational class revealed important trends. However, despite efforts to capture nuances in the probability of WA, the width of the confidence intervals suggests an overlap between men and women in certain groups. This can be ascribed to several factors, such as natural variation in the data or the influence of variables not included in the model. Finally, the nature of the cross-section does not allow establishing causal relationships between the variables analyzed, nor does it make it possible to identify latent variables that could explain gender differences, according to occupational classes. Prospective longitudinal studies are needed to better understand the occurrence of these events.

Once some limitations have been clarified, it is worth mentioning the advantages of the study. Although the gender variable is considered in most research on WA in Brazil^9,11,12^, no results were found focusing on gender differences according to occupational class.

The analysis of the PNS sample made it possible to advance in relation to previous research, which was based on social security data, which are known to be fragile due to deficiencies in official registration systems^4,9,11^. Furthermore, when interviewing workers in their homes, people in different occupations were included, working with or without a formal contract. Thus, it was possible to avoid the limits faced by research focused on a sample of workers employed in a specific sector and regularly hired^9,11,12^.

The occupational class construct, the basis of the investigation that originated the results presented, is essential in research on macrostructural factors related to WA^33^. The analytical lines based on it were valuable in interpreting the probability of the outcome, so as to indicate elements to better understand the inequalities that constitute the occupational structure in our society. In this sense, the presentation of the WA panorama in a representative sample of the Brazilian population covered gaps in knowledge of associated factors. Gender configurations and occupational classes proved to be relevant dimensions in the approach to WA in Brazil.

Deindustrialization is related to the tendency to reduce the prevalence of WA. In several countries, the expansion of services, combined with technological innovations, has changed production processes, forms of work organization and the type of employment. This transformation changed the panorama of occupational environments, explaining, among other factors, the relevance of others risks in certain classes, in light of WA risks^2,5,23^.

The findings are clues for the development of WA prevention actions, with emphasis on gender differences, as well as risk differences related to occupational structure.
