# Supplementary material for: Occupational inequalities and gender differences: work accidents, Brazil, 2019
Source: Rev Saude Publica. 2024 Apr 11;58:13. doi: 10.11606/s1518-8787.2024058005342 (PMC11037903; doi:10.11606/s1518-8787.2024058005342)
Supplement: Translation

# Translation: Desigualdades ocupacionais e diferenças de gênero: acidentes de trabalho, Brasil, 2019

**Authors:** Luciana de Melo Gomides, Mery Natali Silva Abreu, Ada Ávila Assunção

**Affiliations:** I Brasil Universidade Federal de Itajubá. Instituto de Ciências Puras e Aplicadas. Coordenação de Engenharia de Saúde e Segurança. Itabira, MG, Brasil; II Brasil Universidade Federal de Minas Gerais. Faculdade de Medicina. Programa de Pós-Graduação em Saúde Pública. Belo Horizonte, MG, Brasil; III Brasil Universidade Federal de Minas Gerais. Escola de Enfermagem. Departamento de Enfermagem Aplicada. Belo Horizonte, MG, Brasil; IV Brasil Universidade Federal de Minas Gerais. Faculdade de Medicina. Departamento de Medicina Preventiva e Social. Belo Horizonte, MG, Brasil

**Keywords:** Acidentes de Trabalho, Gênero, Fatores Sociodemográficos, Fatores de Risco, Inquéritos Epidemiológicos

## Abstract

**OBJETIVO:**

Analisar a distribuição e associação de fatores sociodemográficos e ocupacionais a acidentes de trabalho (AT) autorrelatados em uma amostra representativa da população brasileira, com ênfase na classe ocupacional, e examinar as diferenças de gênero nessa distribuição.

**MÉTODOS:**

Estudo transversal de base populacional, com dados da Pesquisa Nacional de Saúde (PNS) de 2019, analisou as respostas de uma amostra de adultos com 18 anos ou mais de idade. Fatores associados a AT foram investigados por regressão logística binária e análise hierarquizada por meio de blocos (variáveis sociodemográficas e ocupacionais). O modelo final foi ajustado pelas variáveis de todos os blocos, adotando-se o nível de significância de 5%. Obtiveram-se os valores das razões de chance (RC) e respectivos intervalos de confiança.

**RESULTADOS:**

Entre os participantes, 2,69% relataram ter sofrido AT, sendo mais alta a prevalência em homens (3,37%; IC95% 2,97–3,82%), se comparados às mulheres (1,86%; IC95% 1,55–2,23%). A análise identificou que faixa etária, trabalho noturno, jornada de trabalho e exposição a riscos laborais foram associados a AT, com destaque para as diferenças de gênero. A classe de trabalhadores manuais, tanto qualificados (RC_mulheres_ = 2,87; IC95% 1,33–6,21 e RC_homens_ = 2,46; IC95% 1,37–4,40) quanto não qualificados (RC_mulheres_ = 2,55; IC95% 1,44–4,50 e RC_homens_ = 3,70; IC95% 1,95–7,03), apresentaram maior chance de AT em comparação à classe de gerentes/profissionais.

**CONCLUSÃO:**

Fatores ocupacionais contribuíram significativamente para o aumento na probabilidade de AT para homens e mulheres, com maior magnitude entre aqueles posicionados nos estratos inferiores da estrutura ocupacional. Os resultados obtidos são pistas para a elaboração de ações de prevenção de AT.

## INTRODUÇÃO

Existe convergência entre escolaridade, renda e lugar ocupado pelo indivíduo na estrutura ocupacional que reflete no tipo, resultado e intensidade das atividades desenvolvidas pelos trabalhadores e trabalhadoras. Determinada por processos econômicos e políticos, a estrutura ocupacional representa a ideia de uma sociedade que hierarquiza e discrimina as ocupações^1^. Sabe-se que, nas classes baixas da estrutura, geralmente são maiores as taxas de mortalidade, bem como são piores os resultados de saúde, se comparadas às classes posicionadas no andar de cima^2,3^. Natureza das atividades e condições em que o trabalho é realizado são específicas a cada classe, explicando, ao menos em parte, as desigualdades em saúde^4,5^.

Em primeiro lugar, o acesso ao emprego depende do nível de escolaridade^2^. Em ocupações com menor exigência de escolaridade/qualificação, em segundo, é maior a chance de indivíduos estarem expostos a ambientes insalubres e perigosos, culminando em uma maior prevalência de acidentes de trabalho (AT)^6,7^. De acordo com resultados anteriores, em terceiro lugar, observou-se que os efeitos dos riscos não são idênticos quando há comparação entre os grupos ocupacionais^6^.

AT são eventos súbitos, resultantes de causas não naturais, que ocorrem no ambiente laboral durante o exercício das atividades pelo trabalhador, provocando lesões corporais ou perturbações funcionais. Sendo considerados um problema de saúde pública, são fonte de morbimortalidade e podem gerar incapacidade laborativa e saída precoce da força de trabalho^4,8^. Além de dor e sofrimento para as vítimas, esses eventos geram custos previdenciários, despesas aos serviços de saúde, ônus social e financeiro para toda a sociedade^9,10^.

No âmbito das investigações acerca da distribuição de AT baseadas na estrutura ocupacional, as pesquisas trazem perspectivas esclarecedoras. Embora os resultados recentes no Brasil se concentrem em estudos locais^9,11^, muitos direcionados a categorias específicas^12^, existe possibilidade de uma visão mais abrangente.

Uma abordagem mais completa das desigualdades em AT requer a incorporação das diferenças de gênero nas análises, de forma que é importante esclarecer a conexão entre sexo e gênero, conforme sugerido pelos autores que estudam as articulações entre trabalho e saúde^13^. Sexo diz respeito a fatores biológicos, incluindo cromossômicos e hormonais. Por sua vez, gênero refere-se às expectativas e aos papéis construídos socialmente considerados próprios aos homens ou às mulheres^14^. Esse tipo de atribuição de papéis molda experiências e desigualdades no acesso a recursos e oportunidades. Por exemplo, a distribuição entre homens e mulheres do tempo destinado às atividades de trabalho e domésticas se mantém desequilibrada, em que pese o aumento da participação das mulheres no mercado de trabalho^15^.

Nesse sentido, este estudo teve como objetivo analisar a distribuição e associação de fatores sociodemográficos e ocupacionais ao acidente de trabalho autorrelatado em uma amostra representativa da população brasileira, com ênfase na classe ocupacional, bem como examinar as diferenças de gênero nessa distribuição.

## MÉTODOS

### Desenho do Estudo, Fonte de Dados e População Amostral

O estudo transversal analisou dados secundários da Pesquisa Nacional de Saúde (PNS) – 2019^16,17^. A amostragem probabilística da PNS, representativa da população brasileira com 15 anos ou mais, foi realizada por meio de um processo de conglomerados em três estágios, incluindo a estratificação das unidades primárias de amostragem. No primeiro estágio, foram selecionados os setores censitários, ou conjuntos de setores constitutivos das unidades primárias de amostragem. No segundo estágio, foram selecionados os domicílios. Para o terceiro estágio, em cada domicílio, foi selecionado aleatoriamente um morador para responder ao questionário individual. Detalhes sobre o processo amostral, o desenho e realização da PNS 2019 estão publicados^16^.

Para as análises deste estudo, foram consideradas elegíveis as pessoas ocupadas (entre os moradores selecionados) com idade igual a 18 anos ou mais, que responderam ao módulo do questionário referente à característica do trabalho e apoio social (Módulo M). Foram excluídos militares, empregadores e pessoas que cuidam de afazeres domésticos em casa ou para pessoas próximas. Após a remoção dos inelegíveis, obteve-se uma amostra total de 50.056 indivíduos que responderam a todas as questões de interesse desta pesquisa.

### Variáveis do Estudo

A variável desfecho AT, do tipo dicotômica, foi obtida por meio da resposta à questão O21 do módulo O da PNS 2019 “Nos últimos doze meses o(a) sr.(a) se envolveu em algum acidente de trabalho (sem considerar os acidentes de trânsito e/ou de deslocamento para o trabalho)?”

O primeiro bloco de variáveis independentes diz respeito aos seguintes fatores sociodemográficos: sexo (homem, mulher), faixa etária (18–29 anos, 30–39 anos, 40–49 anos, 50–59 anos e ≥ 60 anos), nível de instrução (superior, médio, fundamental e sem instrução), raça/cor (branca, preta, parda e outras). Os fatores ocupacionais constituem o segundo bloco, sendo os seguintes: trabalho noturno (sim, não), jornada de trabalho semanal (inferior a 40 horas, entre 40 e 44 horas e superior a 44 horas), exposição a riscos laborais, ou seja, a exposição a riscos químicos, físicos ou biológicos no trabalho (sim, não). As classes ocupacionais foram definidas a partir da resposta à questão E12 “Qual era a ocupação (cargo ou função) que tinha nesse trabalho?”.

Classificação de ocupações é um método que permite organizar grupos ocupacionais definidos de acordo com renda, escolaridade, tipo e demanda das tarefas desempenhadas. Indicadores baseados nas classes ocupacionais capturam os efeitos desses fatores sobre a saúde e segurança dos trabalhadores^2,6,18^. A Classificação Brasileira de Ocupações (CBO)^19^ está em linha com a Classificação Internacional Uniformizada das Ocupações (CIUO), elaborada pela Organização Internacional do Trabalho (OIT)^20^ para facilitar a produção de informações sobre o emprego e os trabalhadores de diferentes países. Os critérios de classificação e a terminologia empregada na CIUO para definir e nomear as ocupações são amplamente discutidos e divulgados, de forma a orientar a tomada de decisões e elaborações de ações e programas específicos, bem como apoiar pesquisas^20^. A versão atual da CIUO considera o nível de competência como critério básico para definir o sistema que incorpora grandes grupos ocupacionais, subgrupos principais, subgrupos e grupos de base (famílias ocupacionais). Nesse âmbito, a CBO define competência como “função da complexidade, amplitude e responsabilidade das atividades desenvolvidas no emprego ou outro tipo de relação de trabalho”^19^. A avaliação de uma competência é operacional, pois considera a natureza do trabalho própria àquela ocupação, a escolaridade requerida para desempenhar aquela tarefa, e a formação específica ou experiência prévia, que promoveriam o desenvolvimento das competências pertinentes^20^.

As alternativas de resposta sobre a ocupação que constam do questionário da PNS são baseadas na Classificação de Ocupações para Pesquisas Domiciliares (COD), que é uma adaptação da CBO para as pesquisas domiciliares do Instituto Brasileiro de Geografia e Estatística (IBGE)^21^.

Neste estudo, foi utilizada a composição em seis categorias (Quadro) que representam os dez grandes grupos ocupacionais da COD. Essa estratégia tem a vantagem de evitar excesso de variáveis que acarretaria problemas nas estimativas dos estudos estatísticos^3^. A referida composição foi sugerida na Escala de Status Socioeconômico das Ocupações, desenvolvida por Pastore e Silva^22^, bastante difundida no Brasil por combinar posições educacionais e econômicas individuais dentro de uma classe ocupacional específica. Nessa tipologia, a distinção entre trabalho manual e não manual é uma tentativa de capturar a discriminação resultante da estrutura social e o grau de prestígio conferido às diferentes profissões^1,3,22^.

**Quadro:** Organização das ocupações de acordo com a composição ocupacional de Pastore e Silva^19^ em relação aos grandes grupos ocupacionais e subgrupos da Classificação de Ocupações para Pesquisas Domiciliares (COD) do Instituto Brasileiro de Geografia e Estatística (IBGE)^18^.

Classificação socioeconômica	Reagrupamento GG COD	Composição SG COD
Gerentes e Profissionais de nível superior (Alto)	GG00-Profissionais das forças armadas, bombeiros militares	
	GG01 – Diretores e Gerentes	SG11 – Diretores executivos, Dirigentes da Administração pública e membro do poder executivo
		SG12 – Dirigentes administrativos e comerciais
		SG13 – Dirigentes e gerentes de Produção e Operação
		SG14 – Gerentes de Hotéis, restaurantes comércio e outros serviços
	GG02 – Profissionais das ciências e intelectuais	SG21 – Profissionais das Ciências e da Engenharia
		SG22 – Profissionais da Saúde
		SG23 – Profissionais do Ensino
		SG24 – Especialista em organização da administração
		SG25 – Profissionais de tecnologias da informação e comunicações
		SG26 – Profissionais em direito, em ciências sociais e culturais
Técnicos de nível médio (Médio superior)	GG03 – Técnicos e profissionais de nível médio	SG31 – Profissionais de nível médio das ciências e da engenharia
		SG 32 – Profissionais de nível médio da saúde e afins.
		SG 33 – Profissionais de nível médio em operações financeiras e administrativas.
		SG34 – Profissionais de nível médio de serviços jurídicos, sociais culturais e afins
		SG35 – Técnicos de nível médio da tecnologia da informação e das comunicações.
Trabalhadores não manuais de rotina (médio-médio)	GG 4 – Trabalhadores de apoio administrativo	SG41 – Escriturários
		SG42 – Trabalhadores de atendimento direto ao público
		SG43 – Trabalhadores de cálculo numérico e encarregados do registro de materiais.
		SG44 – Outros trabalhadores de serviços administrativos.
	GG05 – Trabalhadores dos serviços, vendedores do comércio e mercados.	SG51 – Trabalhadores dos serviços pessoais
		SG52 – Vendedores
		SG53 – Trabalhadores dos cuidados pessoais
		SG54 – Trabalhadores dos serviços de proteção e segurança.
Trabalhadores manuais qualificados (médio-inferior)	GG07 – Trabalhadores qualificados, operários e artesãos da construção, das artes mecânicas e outros ofícios.	SG71 – Trabalhadores qualificados e operários da construção inclusive eletricistas.
		SG72 – Trabalhadores qualificados e operários da metalurgia da construção mecânica e afins.
		SG73 – Artesãos e operários das artes gráficas
		SG74 – Trabalhadores especializados em eletricidade e eletrônica
		SG75 – Operários e oficiais de processamento de alimentos da madeira, da confecção e afins.
	GG08 – Operadores de instalações e máquinas e montadores	SG 81 – Operadores de instalações fixas e máquinas
		SG82 – Montadores
		SG83 – Condutores de veículos e operadores de equipamentos móveis pesados
Trabalhadores manuais não qualificados (Baixo-superior)	GG9 – Trabalhadores Elementares	SG91 – Trabalhadores domésticos e outros trabalhadores de limpeza do interior de edifícios.
		SG93 – Trabalhadores elementares da mineração, da construção, da indústria de transformação e do transporte.
		SG94 – Ajudantes de preparação de alimentos
		SG95 – Trabalhadores ambulantes de serviços e afins.
		SG96 – Coletores de lixo e outras ocupações elementares.
Trabalhadores rurais (Baixo-Inferior)	GG09 – Trabalhadores Elementares	SG92 – Trabalhadores elementares da agropecuária, da pesca e florestais.
	GG06 – Trabalhadores qualificados da agropecuária, florestais da caça e da pesca.	SG61 – agricultores qualificados da agropecuária
		SG62 – Trabalhadores Florestais qualificados e caçadores

### Análise Estatística

Todas as análises de AT foram realizadas com atenção às características da amostra complexa da PNS 2019. Dessa feita, foram considerados os fatores de expansão ou pesos amostrais dos domicílios e todos seus moradores, assim como do morador selecionado para a entrevista. Todos oc cálculos foram realizados com auxílio do programa Stata 16.0. Primeiramente, foi realizada análise descritiva da amostra com todas as variáveis de interesse. As diferenças de proporção foram estimadas pelo teste qui-quadrado de Pearson, considerando-se valor de p < 0,05. Foi realizado o cálculo de prevalências com os respectivos intervalos de confiança de 95% (IC95%). A associação entre acidente de trabalho autorreferido com as variáveis independentes foi analisada por meio da regressão logística binária. As análises não ajustada e ajustada foram realizadas com amostra estratificada por sexo.

Foram incluídas na análise ajustada todas as variáveis com valor-p inferior a 0,20 da análise não ajustada. Para o modelo multivariável, foi estabelecido um modelo hierárquico com a inclusão dos dois diferentes blocos: o primeiro composto por variáveis sociodemográficas e, o segundo, por variáveis ocupacionais. Utilizou-se o método *backward* para seleção das variáveis, permanecendo ao final somente aquelas significativas ao nível de 5%. A magnitude da associação foi estimada por meio da razão de chances (RC) com os respectivos IC95% em todas as fases da análise.

A qualidade do ajuste dos modelos foi avaliada por meio do teste de Hosmer-Lemeshow. Foram testadas possíveis interações entre as variáveis que permaneceram no modelo final. Após a definição dos modelos, cálculos preditivos por classe ocupacional foram realizados para homens e mulheres, segundo dois perfis distintos: o primeiro composto pelas características de maior chance de AT e o segundo com as características de menor chance.

## RESULTADOS

Dois milhões e quarenta e oito mil trabalhadores brasileiros com 18 anos ou mais de idade relataram ter sofrido AT entre 2018 e 2019, o que corresponde a 2,69% (IC95% 2,42–2,98%) da amostra analisada. A maior prevalência foi observada entre os homens se comparados às mulheres, 3,37% (IC95% 2,97–3,82%) e 1,86% (IC95% 1,55–2,23%), respectivamente. Foram observadas diferenças na prevalência de AT para as variáveis, de acordo com o gênero (Tabela 1).

**Tabela 1:** Proporções de homens e mulheres e prevalências de acidentes de trabalho estratificadas por sexo, de acordo com as características sociodemográficos e ocupacionais. Pesquisa Nacional de Saúde, Brasil, 2019.

Variáveis	Homens			Mulheres			valor de p^a^
	
	(n_exp_ = 50.541.462)			(n_exp_ = 41.991.625)			
	
	n_exp_/1000	%	Prevalência acidentes	n_exp_/1000	%	Prevalência acidentes	
	
			(IC95%)			(IC95%)	
Acidente de trabalho							< 0,001
Não	48.837,17	96,63	-	41.211,19	98,14		
Sim	1.704,30	3,37	3,37 (2,97–3,82)	780,43	1,86	1,86 (1,55–2,23)	
Faixa etária (anos)							< 0,001
≥ 60	12.244,81	9,15	2,16 (1,48–3,13)	9.624,72	7,86	0,58 (0,34–1,01)	
50–59	9.453,33	18,7	2,90 (2,16–3,87)	7.234,05	17,23	2,02 (1,39–2,94)	
40–49	10.767,98	21,31	3,13 (2,18–4,47)	10.304,56	24,54	2,26 (1,71–2,98)	
30–39	13.450,26	26,61	3,50 (2,79–4,39)	11.526,14	27,45	1,47 (1,06–2,04)	
18–29	4.625,08	24,23	4,27 (3,37–5,39)	3.302,15	22,92	2,21 (1,40–3,48)	
Raça/cor							< 0,001
Branca	20.971,49	41,49	2,75 (2,24–3,38)	18.866,19	44,93	1,55 (1,11–2,17)	
Preta	6.107,36	12,08	4,41 (3,28–5,91)	5.138,69	12,24	2,25 (1,30–3,86)	
Parda	22.649,09	44,81	3,74 (3,10–4,51)	17.465,47	44,81	2,11 (1,67–2,66)	
Outros^b^	813,52	1,61	1,21 (0,59–2,45)	521,27	1,24	0,67 (0,25–1,76)	
Nível de instrução							< 0,001
Superior	7.583,61	15	1,22 (0,74–1,99)	10.238,29	24,38	1,17 (0,79–1,72)	
Ensino médio	19.026,78	37,65	2,95 (2,43–3,58)	17.560,47	41,82	2,29 (1,71–3,06)	
Fundamental	8.450,14	16,72	4,52 (3,15–6,45)	5.255,97	12,52	1,94 (1,34–2,79)	
Sem instrução	15.480,93	30,63	4,32 (3,62–5,14)	8.936,90	21,28	1,75 (1,26–2,43)	
Classe ocupacional							< 0,001
Gerentes/profissionais	5.682,31	11,24	1,01 (0,60–1,71)	7.329,41	17,45	0,97 (0,62–1,51)	
Técnicos	4.194,52	8,3	1,52 (1,01–2,29)	3.340,87	7,96	2,18 (1,38–3,43)	
Não manual de rotina	11.527,50	22,81	2,05 (1,46–2,85)	17.316,08	41,24	1,62 (1,16–2,26)	
Manual qualificado	16.757,40	33,16	3,9 (3,27–4,64)	3.639,55	8,67	3,00 (1,60–5,55)	
Rural	6.675,94	13,21	5,44 (3,86–7,62)	1.643,65	3,91	1,66 (0,97–2,84)	
Manual não qualificado	5.703,79	11,29	5,80 (4,24–7,88)	8.722,08	20,77	2,51 (1,83–3,45)	
Trabalho noturno							< 0,001
Não	42.496,53	84,08	3,2 (2,77–3,69)	37.930,93	90,33	1,72 (1,40–2,10)	
Sim	8.044,93	15,92	4,29 (3,34–5,50)	4.060,70	9,67	3,17 (2,17–4,61)	
Jornada semanal (horas)							< 0,001
< 40	25.314,06	20,54	1,96 (1,56–2,46)	27.766,83	39,24	1,26 (0,89–1,77)	
40–44	10.861,41	51,04	3,34 (2,86–3,90)	6.887,88	43,29	2,23 (1,69–2,94)	
> 44	14.365,99	28,42	4,46 (3,50–5,66)	7.336,92	17,47	2,28 (1,71–3,02)	
Exposição ao risco laboral							< 0,001
Não	19.932,25	39,44	1,12 (0,81–1,54)	27.014,29	64,33	0,91 (0,69–1,19)	
Sim	30.609,22	60,56	4,84 (4,22–5,54)	14.977,33	35,67	3,57 (2,83–4,49)	

N_exp_: número expandido; IC95%: intervalo de confiança de 95%.

Nota: percentual por gênero da amostra: homens, 54,62% (IC95% 53,76–55,47%) e mulheres 45,38% (IC95% 44,53–46,24%).

^a^ Associado ao teste de igualdade de proporções baseado na estatística qui-quadrado de Pearson.

^b^ Outros: pessoas que se autodeclararam amarelos e indígenas.

Na análise não ajustada, todas as variáveis independentes foram mantidas (p < 0,20) e, portanto, levadas para análise ajustada para ambos os sexos (Tabelas 2 e 3). No modelo final ajustado para as mulheres, as variáveis faixa etária, classes ocupacionais, trabalho noturno, jornada e exposição a riscos laborais permaneceram associadas ao desfecho (Tabela 2). Observaram-se maiores chances de AT entre trabalhadoras posicionadas na classe manual qualificada (RC = 2,87; IC95% 1,33–6,21) quando comparadas à classe de gerentes e profissionais. Maiores chances de AT foram verificadas entre as mulheres com autorrelato de jornada entre 40 e 44 horas semanais (RC = 1,87; IC95% 1,20–2,90) e jornada superior a 44 horas semanais (RC = 1,58; IC95% 1,00–2,49), se comparadas às mulheres que autorrelataram jornada inferior a 40 horas semanais. O modelo identificou maiores chances de AT entre aquelas que afirmaram realizar trabalho noturno (RC = 1,59; IC95% 1,00–2,56). Vale destacar a interação entre faixa etária e exposição a riscos laborais, provocando a necessidade de produzir um modelo adicional para melhor discriminar os resultados (Tabela 4).

**Tabela 2:** Estimativas não ajustadas e ajustadas de razões de chances de acidente de trabalho, entre mulheres, associadas às características sociodemográficas e ocupacionais, Pesquisa Nacional de Saúde, Brasil, 2019.

Variáveis	n_exp_/1000	Não ajustado		Ajustado		
	
		RC	valor de p	Bloco 1 Sociodemográficos	Bloco 2 Ocupacionais	Modelo final (Bloco 1+2)
		
				RC (IC95%)	RC (IC95%)	RC (IC95%)
Faixa etária (anos)						
≥ 60	9.624,72	Ref.		Ref.	-	-
50–59	7.234,05	3,53	< 0,001	3,56 (1,81–6,97)	-	-
40–49	10.304,56	3,95	< 0,001	3,95 (2,11–7,41)	-	-
30–39	11.526,14	2,55	0,005	2,54 (1,30–4,96)	-	-
18–29	3.302,15	3,86	< 0,001	3,54 (1,73–7,24)	-	-
Raça/cor						
Branca	44,93	Ref.		-	-	-
Preta	12,24	1,46	0,268	-	-	-
Parda	44,81	1,36	0,142	-	-	-
Outros^a^	1,24	0,43	0,108	-	-	-
Nível de instrução						
Superior	10.238,29	Ref.		Ref.	-	-
Ensino médio	17.560,47	1,98	0,007	1,94 (1,21–3,12)	-	-
Fundamental	5.255,97	1,67	0,064	1,64 (0,95–2,83)	-	-
Sem instrução	8.936,90	1,5	0,121	1,57 (0,93–2,64)	-	-
Classe ocupacional						
Gerentes/profissionais	7.329,41	Ref.		-	Ref.	Ref.
Técnicos	3.340,87	2,27	0,012	-	1,60 (0,84–3,03)	1,64 (0,86–3,11)
Não manual de rotina	17.316,08	1,68	0,068	-	1,74 (0,98–3,07)	1,71 (0,98–2,98)
Manual qualificado	36.39,55	3,15	0,004	-	2,86 (1,32–6,20)	2,87 (1,33–6,21)
Rural	1.643,65	1,72	0,133	-	1,26 (0,60–2,63)	1,36 (0,65–2,86)
Manual não qualificado	8.722,08	2,63	0,001	-	2,48 (1,40–4,38)	2,55 (1,44–4,50)
Trabalho noturno						
Não	37.930,93	Ref.		-	Ref.	Ref.
Sim	4.060,70	1,87	0,005	-	1,60 (1,00–2,53)	1,59 (1,00–2,56)
Jornada (horas)						
< 40	27.766,83	Ref.		-	Ref.	Ref.
40–44	6.887,88	1,8	0,01	-	1,85 (1,19–2,86)	1,87 (1,20–2,90)
> 44	7.336,92	1,83	0,008	-	1,60 (1,02–2,53)	1,58 (1,00–2,49)
Exposição ao risco laboral						
Não	27.014,29	Ref.		-	Ref.	-
Sim	14.977,33	4,03	< 0,001	-	3,79 (2,58–5,58)	-
Faixa etária (anos) #riscos laborais						
50–59 #riscos (sim)	2.445,40	-	-	-	-	-
40–49 #riscos (sim)	4.016,07	-	-	-	-	-
30–39 #riscos (sim)	4.452,58	-	-	-	-	-
18–29 #riscos (sim)	908,36	-	-	-	-	-

Ref.: categoria de referência; RC: razão de chance*;* IC95%: intervalo de confiança de 95%; n_exp_ = número expandido.

Valor-p do teste de Hosmer-Lemeshow modelo final: 0,96.

^a^ Outros: pessoas que se autodeclararam amarelos e indígenas.

**Tabela 4:** Análise multivariada avaliando fatores associados ao uso de risco de AT em mulheres, estratificando por exposição ao risco laboral, segundo a PNS, 2019.

Variáveis	RC (IC95%)	

	Mulheres sem exposição ao risco laboral	Mulheres com exposição ao risco laboral
Faixa etária (anos)		
≥ 60	Ref.	Ref.
50–59	2,83 (1,16–6,93)	3,40 (1,28–9,00)
40–49	3,53 (1,38–9,05)	3,19 (1,31–7,80)
30–39	1,91 (0,63–5,73)	2,25 (0,92–5,51)
18–29	1,06 (0,39–2,91)	5,55 (2,16–14,30)
Classe ocupacional		
Gerentes/profissionais	Ref.	Ref.
Técnicos	1,45 (0,41–5,17)	1,62 (0,80–3,28)
Não manual de rotina	1,87 (0,70–4,98)	1,62 (0,83–3,13)
Manual qualificado	1,92 (0,45–8,19)	3,31 (1,40–7,84)
Rural	3,71 (0,75–1,83)	1,07 (0,50–2,26)
Manual não qualificado	2,81 (0,98–8,05)	2,42 (1,28–4,58)
Trabalho noturno		
Não	Ref.	Ref.
Sim	0,88 (0,38–2,05)	1,84 (1,05–3,24)
Jornada semanal (horas)		
< 4	Ref.	Ref.
40–44	2,17 (1,12–4,21)	1,72 (0,97–3,06)
> 44	2,19 (0,98–4,91)	1,35 (0,78–2,34)

AT: acidente de trabalho; RC: razão de chance; IC95%: intervalo de confiança de 95%; Ref.: categoria de referência.

No modelo adicional (Tabela 4) estratificado por exposição a riscos laborais, observou-se, no grupo de mulheres expostas, maior chance de AT entre aquelas com idade entre 18 e 29 anos (RC = 5,55; IC95% 2,16–14,30), se comparadas às mulheres com idade superior a 60 anos.

Entre os homens, o modelo final ajustado (Tabela 3) indicou maior chance de AT no grupo de trabalhadores manuais não qualificados (RC = 4,23; IC95% 2,24–8,00), se comparados ao grupo de referência. Observou-se que o aumento na duração da jornada de trabalho, no grupo dos homens, está relacionado ao aumento da chance de AT. Autorrelato de trabalho noturno (RC = 1,47; IC95% 1,08–2,00) e exposição a riscos laborais (RC = 3,37; IC95% 2,40–4,74) foram associados ao desfecho. Uma chance maior de AT foi observada no grupo de trabalhadores na faixa etária entre 18 e 29 anos (RC = 1,84; IC95% 1,15–2,94), se comparado ao grupo com idade superior a 60 anos. Os modelos para ambos os sexos apresentaram bom ajuste, segundo a estatística de Hosmer-Lemeshow (p > 0,05).

**Tabela 3:** Estimativas não ajustadas e ajustadas de razões de chances de acidente de trabalho, entre homens, associadas às características sociodemográficas e ocupacionais, Pesquisa Nacional de Saúde, Brasil, 2019.

Variáveis	n_exp_/1000	Não ajustado		Ajustado		
	
		RC	Valor de p	Bloco 1 Sociodemográficos	Bloco 2 Ocupacionais	Modelo final (Bloco 1+2)
		
				RC (IC95%)	RC (IC95%)	RC (IC95%)
Faixa etária (anos)						
≥ 60	12.244,81	Ref.		Ref.		Ref.
50–59	9.453,33	1,35	0,224	1,41 (0,87–2,29)		1,19 (0,73–1,95)
40–49	10.767,98	1,46	0,158	1,61 (0,96–2,71)		1,25 (0,73–2,17)
30–39	13.450,26	1,65	0,028	2,01 (1,27–3,20)		1,42 (0,91–2,23)
18–29	4.625,08	2,02	0,003	2,37 (1,47–3,80)		1,84 (1,15–2,94)
Raça/cor						
Branca	20.971,49	Ref.		-		-
Preta	6.107,36	1,63	0,007	-		-
Parda	22.649,09	1,37	0,034	-		-
Outros^a^	813,52	0,43	0,027	-		-
Nível de instrução						
Superior	7.583,61	Ref.		Ref.		-
Ensino médio	19.026,78	2,47	0,001	2,27 (1,32–3,88)		-
Fundamental	8.450,14	3,84	< 0,001	3,57 (1,88–6,78)		-
Sem instrução	15.480,93	3,66	< 0,001	4,01 (2,32–6,95)		-
Classe ocupacional						
Gerentes/profissionais	5.682,31	Ref.			Ref.	Ref.
Técnicos	4.194,52	1,51	0,233		1,21 (0,61–2,40)	1,17 (0,59–2,33)
Não manual de rotina	11.527,50	2,04	0,027		1,89 (1,00–3,56)	1,80 (0,96–3,37)
Manual qualificado	16.757,40	3,97	< 0,001		2,52 (1,40–4,52)	2,46 (1,37–4,40)
Rural	6.675,94	5,64	< 0,001		3,64 (1,88–7,07)	3,63 (1,87–7,04)
Manual não qualificado	5.703,79	6,02	< 0,001		4,03 (2,12–7,70)	3,70 (1,95–7,03)
Trabalho noturno						
Não	42.496,53	Ref.			Ref.	Ref.
Sim	8.044,93	1,36	0,044		1,50 (1,10–2,04)	1,47 (1,08–2,00)
Jornada (horas)						
< 40	25.314,06	Ref.			Ref.	Ref.
40–44	10.861,41	1,73	< 0,001		1,74 (1,31–2,31)	1,73 (1,31–2,30)
> 44 horas	14.365,99	2,33	< 0,001		2,17 (1,48–3,17)	2,19 (1,51–3,20)
Exposição ao risco laboral						
Não	19.932,25	Ref.			Ref.	Ref.
Sim	30.609,22	4,49	< 0,001		3,36 (2,38–4,74)	3,37 (2,40–4,74)

RC: razão de chance; IC95%: intervalo de confiança de 95%; Ref.: categoria de referência; n_exp_ = número expandido.

Valor-p do teste de Hosmer-Lemeshow modelo final: 0,93.

^a^ Outros: pessoas que se autodeclararam amarelos e indígenas.

As probabilidades de AT para as classes ocupacionais do grupo de mulheres foram analisadas (Figura A) de acordo com os seguintes perfis: 1) idade de 60 anos ou mais sem exposição a riscos no ambiente laboral, sem relato de trabalhar à noite, jornada de trabalho inferior a 40 horas semanais; 2) idade entre 18 e 29 anos com exposição a riscos no ambiente laboral, autorrelato de trabalhar à noite, jornada de trabalho entre 40 e 44 horas semanais. Para as mulheres gerentes ou profissionais do perfil 1, a probabilidade de relatar acidentes foi 0,14% (IC95% 0,01–0,28%), sendo que do perfil 2, a probabilidade foi 5,95% (IC95% 1,97–9,93%), ainda que inseridas na mesma classe ocupacional. No perfil 1, as probabilidades do desfecho entre as mulheres das classes manual qualificada, rural e manual não qualificada foram, respectivamente, 0,41% (IC95% 0,02–0,89%), 0,19% (IC95% 0,00–0,39%) e 0,36% (IC95% 0,05–0,68%). Quando avaliadas as mulheres de perfil 2, aumentou em 15,36% (IC95% 4,30–26,42%) a probabilidade de AT na classe manual qualificada, 7,93% (IC95% 1,85–14,01%) na classe rural e 13,87% (IC95% 5,21–22,52%) na classe manual não qualificada (Figura A), se comparadas às mulheres do perfil 1.

**Figura:** Probabilidade estimada de ocorrência de acidente de trabalho para mulheres (A) e homens (B) de acordo com classes ocupacionais. Pesquisa Nacional de Saúde, Brasil, 2019.

AT: acidente de trabalho.

Em relação às classes ocupacionais (Figura B), para os homens foram estudados os seguintes perfis: 1) idade 60 anos ou mais, sem relato de trabalhar à noite, jornada inferior a 40 horas semanais, e sem exposição a riscos laborais; 2) faixa etária entre 18 e 29 anos, autorrelato de trabalhar à noite, jornada superior a 44 horas semanais e exposição a riscos laborais. Para os homens gerentes ou profissionais do perfil 1, a probabilidade de relatar acidentes foi de 0,23% (IC95% 0,06–0,39%), enquanto no perfil 2, inseridos na mesma classe, a probabilidade foi de 4,36% (IC95% 1,79–6,92%). A probabilidade de AT entre os homens das classes manual qualificada, rural e manual não qualificada do perfil 1 foi 0,56% (IC95% 0,22–0,90%), 0,82% (IC95% 0,35–1,29%) e 0,84% (IC95% 0,31–1,37%), respectivamente. Quando avaliados os homens de perfil 2, a probabilidade de AT aumentou em 10,06% (IC95% 6,78–13,34%) na classe manual qualificada, 14,17% (IC95% 7,88–20,86%) na classe rural e 14,42% (IC95% 8,48–20,36%) na classe manual não qualificada (Figura B), se comparados aos homens do perfil 1.

## DISCUSSÃO

Pela primeira vez, são apresentados resultados referentes à população brasileira sobre a chance de AT em homens e em mulheres, de acordo com as classes ocupacionais. Seguindo a tendência mundial^23^, observou-se diminuição da prevalência de AT, em 2019, se comparada à edição anterior da PNS^10^.

Além disso, há maior chance de AT em homens e mulheres posicionados nas ocupações classificadas como predominantemente manuais. Essas ocupações são caracterizadas por tarefas de conteúdo do tipo rotineiro ou elementar; ou seja, atividades repetitivas e predefinidas, para as quais não há exigência de qualificação especializada. De acordo com as classificações padronizadas supracitadas, as ocupações predominantemente manuais são posicionadas no andar de baixo da hierarquia ocupacional^1,19^. Trabalhadores nessas ocupações, geralmente, são menos escolarizados, raramente treinados nos temas relativos à proteção e segurança ocupacional, e usufruem de menor poder para evitar a exposição a riscos de AT^7^.

Convergente com a literatura, respondentes mais jovens apresentaram maior chance de relatar AT. Primeiramente porque o mercado de trabalho seleciona indivíduos com maior capacidade muscular e habilidade sensorial para ocupar os postos mais inseguros e insalubres. Aqueles que não sofreram os efeitos da exposição laboral ou das intempéries da vida, geralmente mais jovens, terão mais oportunidades nesse tipo de processo seletivo^24,25^. O denominado efeito trabalhador sadio expressa tal viés de seleção, ou seja, os que perderam a capacidade para enfrentar as exigências de força, vigilância e agilidade não “sobreviveram” no mercado de trabalho, pois estão doentes ou se aposentaram mais cedo^25^. Em segundo lugar, os mais jovens ainda não tiveram experiência suficiente para desenvolver habilidades de autoproteção, explicando, ao menos em parte, a maior chance de AT nessa faixa etária^26^.

Os resultados referentes a jornadas prolongadas e trabalho noturno foram estatisticamente significativos em ambos os sexos. Os mecanismos pelos quais esses fatores afetam negativamente a saúde e a segurança ocupacional estão bem documentados^27^. Alteração das funções cognitivas e aumento da fadiga são efeitos das perturbações do ciclo circadiano, que ocorrem quando as pessoas trabalham à noite e também são esperados quando o tempo para recuperação é reduzido em face das extensas jornadas. Esses efeitos são condições na origem dos AT^28^.

A análise das probabilidades de AT nas classes ocupacionais mostra a associação significativa da idade e dos fatores ocupacionais na ocorrência desses eventos. Observou-se que idade mais jovem, exposição a riscos laborais, trabalho noturno e jornadas prolongadas, quando combinados, contribuem para o aumento expressivo das probabilidades de AT em todas as classes ocupacionais, tanto para homens quanto para as mulheres.

As diferenças nas probabilidades de AT entre as distintas classes se destacaram, principalmente no grupo masculino, quando avaliado o perfil 2. As classes de trabalhadores rurais e manuais não qualificados, sujeitas às condições associadas a uma maior chance de AT, exibiram percentuais significativamente mais elevados em comparação com as classes de gerentes e profissionais quando expostos ao mesmo conjunto de fatores. Esse resultado é consistente com a hipótese inicial deste estudo.

As diferenças de gênero observadas são passíveis de interpretação^14^, indicando elementos para investigações mais aprofundadas. Sabe-se que as exposições ocupacionais podem variar quando as mulheres são comparadas aos homens da mesma classe, porque, geralmente, elas desempenham tarefas que não são atribuídas a eles dentro de uma mesma ocupação^29^. Por exemplo, nos estabelecimentos hospitalares, os auxiliares de enfermagem estão expostos a tarefas que solicitam movimentação de carga (movimentar paciente no leito etc.), com mais chance de queda ou torções de tronco, e as auxiliares se responsabilizam pelos procedimentos que exigem mais destreza, como empacotar materiais ou preparar medicações, com maior chance de dores nos membros superiores^5,29^.

Mesmo com o aumento do número de mulheres no mercado de trabalho nas últimas décadas em países industrializados, as normas de gênero continuam a estruturar a divisão sexual do trabalho^5,14,29^. Tarefas domésticas e cuidado da família são papéis desigualmente distribuídos na sociedade. Sendo que, no caso das mulheres, aos efeitos dos riscos experimentados durante a jornada profissional, somam-se os prejuízos da restrição do tempo de recuperação, entre outros problemas relacionados ao tempo dedicado ao trabalho doméstico. Embora historicamente sub-representadas em trabalhos considerados mais perigosos, as mulheres passaram a ocupar postos anteriormente dominados pelo sexo masculino, por exemplo, a construção civil^30^. Essa realidade atual pode ser uma hipótese para explicar o aumento da probabilidade de AT quando as mulheres estão expostas a riscos ocupacionais que são bem definidos nos setores maciçamente masculinos. Nesses casos, além dos riscos ocupacionais específicos a cada setor produtivo, pesaram sobre as trabalhadoras os estereótipos de gênero no ambiente ocupacional. Ou seja, os referidos estereótipos explicariam tanto o menor poder para interferir no seu próprio ambiente quanto o menor acesso das mulheres aos cargos de direção^31^. Por último, vale mencionar que as pesquisas de gênero alertam sobre o design fortemente masculino das instalações e equipamentos, sem consideração às especificidades biológicas de sexo^5,29^.

As interpretações dos resultados obtidos esbarram em algumas limitações. É possível que viés de memória tenha provocado subestimação dos resultados. Contudo, o desfecho é considerado menos sensível ao esquecimento. Fatores psicossociais, condições de saúde e hábitos de vida, que podem influenciar a chance de AT, não foram abordados. No entanto, há evidências de que, quando relacionados ao AT, a contribuição desses fatores é limitada para a explicação das discrepâncias observadas se comparados às características ocupacionais^6,32^. Alta prevalência de AT foi observada entre os homens da classe rural. No entanto, o resultado não alcançou significância estatística entre as mulheres dessa classe, tornando inviável uma análise aprofundada. Vale destacar um possível viés de representatividade, uma vez que o pequeno número de AT observado no grupo das trabalhadoras rurais pode ter influenciado o poder do estudo em identificar associações significativas nessa classe ocupacional.

As diferenças de gênero na probabilidade de AT por classes ocupacionais revelaram importantes tendências. No entanto, apesar dos esforços para capturar nuances na probabilidade de AT, a amplitude dos intervalos de confiança sugere uma sobreposição entre homens e mulheres em certos grupos. Isso pode ser atribuído a diversos fatores, como a variação natural nos dados ou a influência de variáveis não incluídas no modelo. Por fim, a natureza do corte transversal não autoriza estabelecer relações de causalidade entre as variáveis analisadas, tampouco viabiliza identificar variáveis latentes que poderiam explicar as diferenças de gênero, de acordo com as classes ocupacionais. Estudos longitudinais prospectivos são necessários para melhor compreensão da ocorrência desses eventos.

Uma vez esclarecidas algumas limitações, cabe mencionar as vantagens do estudo. Embora a variável sexo seja considerada na maioria das pesquisas sobre AT no Brasil^9,11,12^, não foram encontrados resultados com foco nas diferenças de gênero de acordo com a classe ocupacional.

## CONCLUSÃO

A análise da amostra da PNS permitiu avançar em relação a pesquisas anteriores, que foram baseadas em dados previdenciários, reconhecidamente frágeis em razão de deficiências dos sistemas oficiais de registro^4,9,11^. Além disso, ao entrevistar trabalhadores em seus domicílios, foram incluídas pessoas inseridas em distintas ocupações, trabalhando com ou sem contrato formal. Assim, foi possível evitar os limites enfrentados pelas pesquisas focalizadas em amostra de trabalhadores empregados num setor específico e regularmente contratados^9,11,12^.

O construto classe ocupacional, base da investigação que originou os resultados apresentados, é essencial na pesquisa sobre os fatores macroestruturais relacionados a AT^33^. As linhas analíticas calcadas nele foram valiosas para interpretar a probabilidade do desfecho, de maneira a indicar elementos para melhor compreender as desigualdades que constituem a estrutura ocupacional em nossa sociedade. Nesse sentido, a apresentação do panorama dos AT em uma amostra representativa da população brasileira cobriu lacunas quanto ao conhecimento dos fatores associados. Configurações de gênero e classes ocupacionais mostraram-se dimensões relevantes na abordagem dos AT no Brasil.

A desindustrialização tem relação com a tendência de diminuição da prevalência de AT. Em vários países, a expansão dos serviços, combinada às inovações tecnológicas, modificou os processos produtivos, as formas de organização do trabalho e o tipo de emprego. Essa transformação alterou o panorama dos ambientes ocupacionais, explicando, entre outros fatores, a relevância de outros riscos em determinadas classes, em face dos riscos de AT^2,5,23^.

Os resultados obtidos são pistas para a elaboração de ações de prevenção de AT, com destaque para os recortes de gênero, bem como para as diferenças de risco relacionadas à estrutura ocupacional.
